# Tracheobronchial Involvement of Rosai–Dorfman Disease: Case Report and Review of the Literature

**DOI:** 10.1097/MD.0000000000002821

**Published:** 2016-02-18

**Authors:** Louis Boissière, Martine Patey, Olivier Toubas, Juliette Vella-Boucaud, Jeanne-Marie Perotin-Collard, Gaëtan Deslée, Francois Lebargy, Sandra Dury

**Affiliations:** From the Department of Respiratory Diseases (LB, JV-B, J-MP-C, GD, FL, SD); Department of Pathology (MP); Radiology Department (OT); INSERM UMRS 903, Reims University Hospital (J-MP-C, GD); and EA 4683 Medical and Pharmacological University of Reims (FL, SD), Reims, France.

## Abstract

Rosai–Dorfman Disease (RDD) is a rare non-neoplastic entity, also known as sinus histiocytosis with massive lymphadenopathy (SHML), characterized by a benign proliferation of histiocytes in lymph nodes. Localized forms of RDD involving the tracheobronchial tree are very rare. There is no consensus regarding the management of central airway forms and recurrence is frequent.

We report the case of an 81-year-old Caucasian woman admitted in 2014 for chronic cough. Her main medical past history included a diagnosis of sinonasal RDD in 1996 with recurrent obstructive rhinosinusitis requiring repeated sinonasal surgery, and a diagnosis of tracheal RDD in 2010 with 2 asymptomatic smooth lesions (5 and 7 mm) on the anterior tracheal wall. Physical examination was normal in 2014. Pulmonary function tests showed an obstructive pattern. Computed tomographic scan revealed a mass arising from the anterior wall of the trachea that projects into the tracheal lumen. Fiberoptic bronchoscopy showed a hypervascular multilobular lesion (2 cm) arising from the anterior tracheal wall and causing 50% obstruction of the tracheal lumen. Mechanical resection with electrocoagulation of the tracheal mass was performed by rigid bronchoscopy with no complication. Histological examination demonstrated tracheal RDD. One year after endotracheal resection, the patient presented no recurrence of cough and the obstructive pattern had resolved.

Reports on tracheobronchial involvement are scarce. Symptomatic tracheobronchial obstruction requires mechanical resection by rigid bronchoscopy or surgery. Recurrence is frequent, justifying long-term follow-up.

## INTRODUCTION

Rosai–Dorfman Disease (RDD) is a rare non-neoplastic entity, also known as sinus histiocytosis with massive lymphadenopathy (SHML), characterized by a benign proliferation of histiocytes in lymph nodes. Painless cervical adenopathy (87%) and impairment of general condition are the major symptoms.^1^ Other lymph node groups may be affected and extranodal involvement is reported in 43% of cases.^1^ Intrathoracic manifestations are described in only 2% of patients^1^ including hilar or mediastinal lymphadenopathy, pulmonary nodules or masses and rarely pleural effusion, interstitial lung disease, or central airway involvement.^1,2^ We report a case of recurrent tracheal involvement in an 81-year-old woman in whom RDD was initially confined to the nasal cavity and paranasal sinuses, and review the 11 cases previously published in the English language literature.

## CASE REPORT

In 1996, a 63-year-old Caucasian woman was admitted to the hospital with sinus obstruction related to thickening and polypoid growth of the mucosa. Her medical history included ischemic heart disease and hypercholesterolemia. Treatment included lysine acetylsalicylate, celiprolol, and simvastatin. Histological examination of right and left nasal mucosa showed a histiocytic proliferation highly suggestive of RDD (CD68+, PS100+, CD1a- with emperipolesis, polyclonal plasmocytosis with no pathogenic agent on PAS and Ziehl staining). From 1998 to 2003, several sinonasal surgical procedures were performed for recurrent sinonasal obstruction.

In 2010, the patient (77 years) presented with isolated cough. Pulmonary function tests were normal. Computed tomography (CT) scan showed 2 nodules protruding into the tracheal lumen. Neither intrathoracic nor cervical lymphadenopathy was observed. Fiberoptic bronchoscopy showed the presence of 2 smooth lesions on the anterior tracheal wall close to the origin of the right main bronchus (7 mm) and above the carina (5 mm). Histological examination of both biopsy samples concluded on a diagnosis of RDD. Cough resolved after discontinuation of perindopril that had been recently prescribed. The patient remained asymptomatic from 2010 to 2014.

**FIGURE 1:**
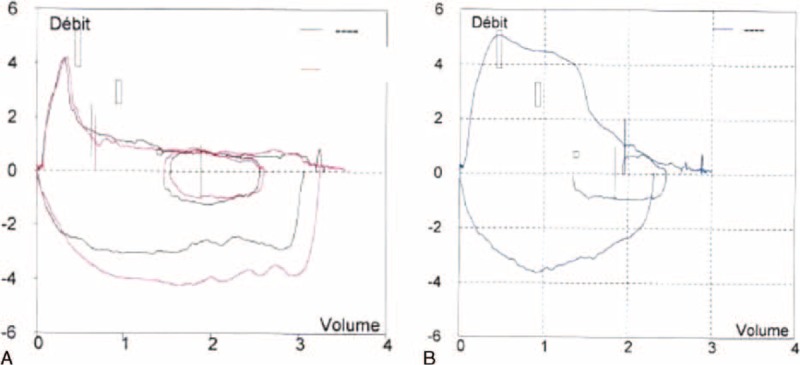
Flow volume loop before mechanical resection of RDD tracheal lesion (red curve after bronchodilators) (A) and after mechanical resection of the tracheal lesion (B).

**FIGURE 2:**
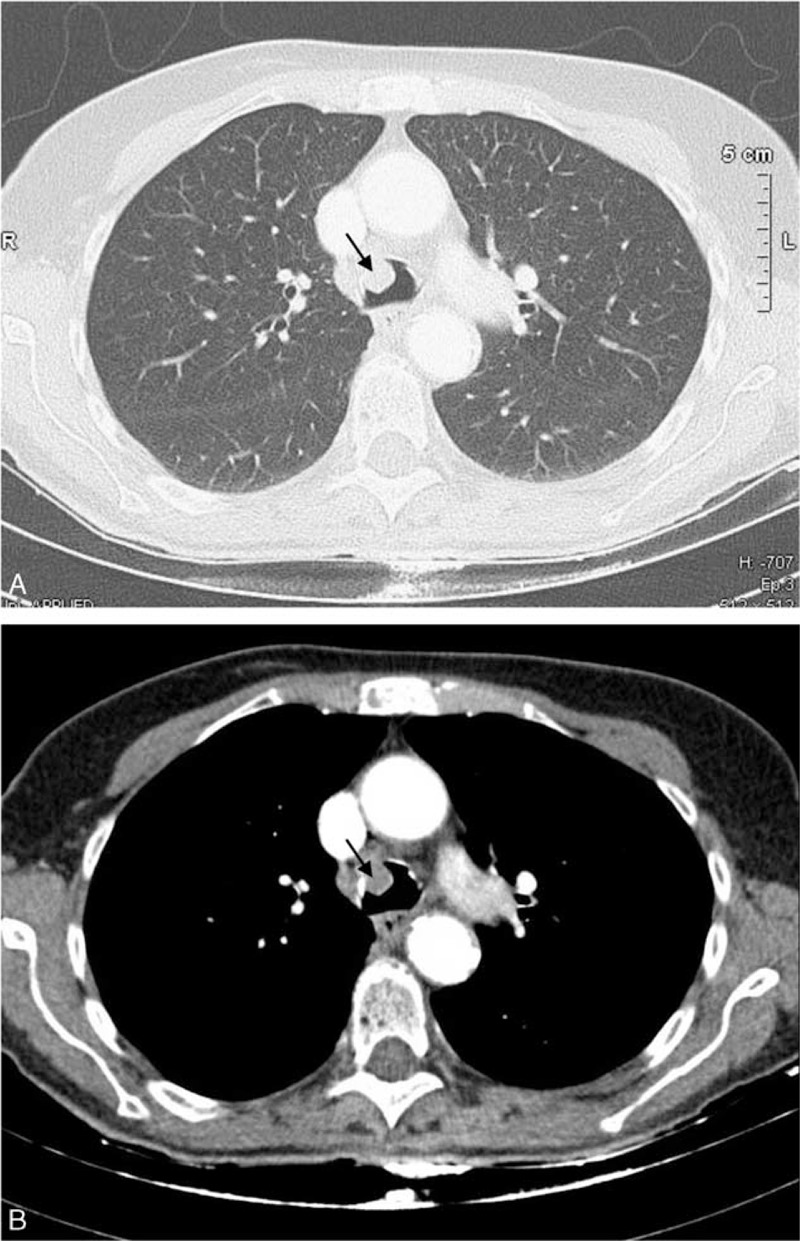
Chest computed tomography (A, Lung window; B, Soft tissue window): endotracheal mass.

**FIGURE 3:**
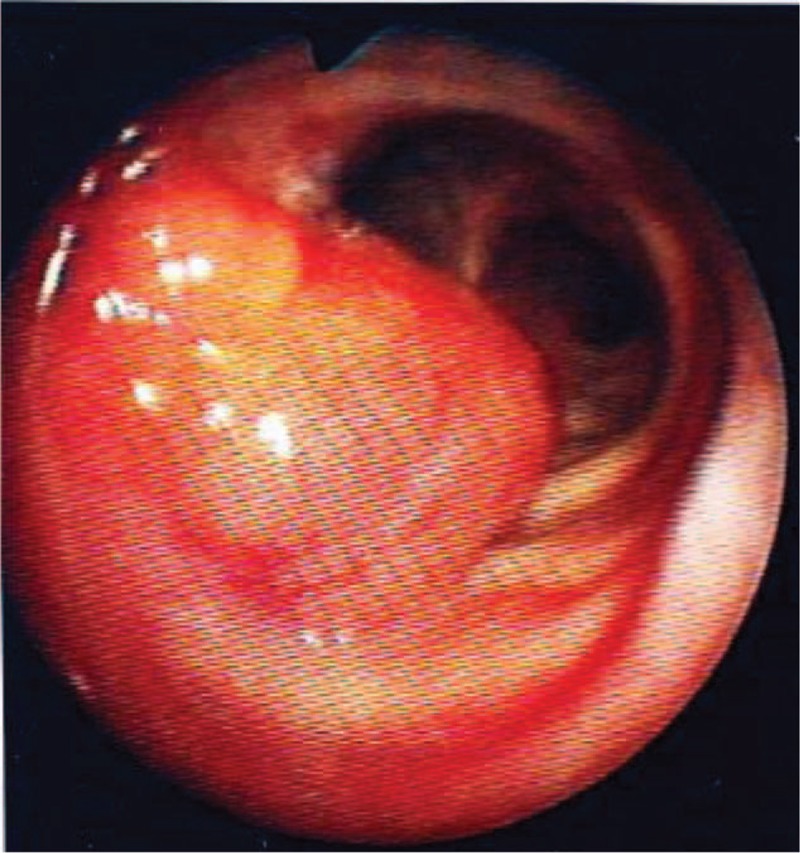
Intratracheal mass on bronchoscopy.

**FIGURE 4:**
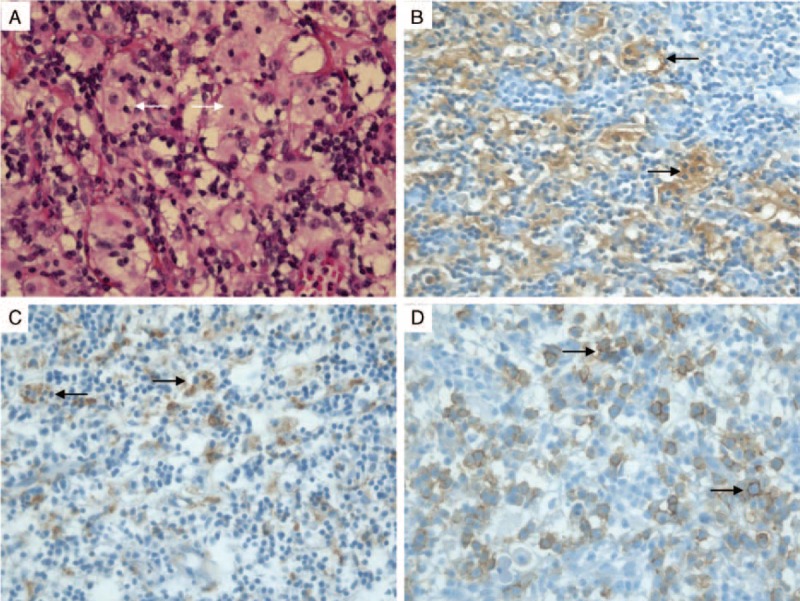
Histological findings. Infiltration of the tracheal mucosa by histiocytes with engulfment of lymphocytes (emperipolesis) (A). Immunolabeling for S-100 protein (B) and CD68 (C) in macrophages; CD138 expression for plasmocytes (D). Magnification ×400.

In 2014, at the age of 81 years, the patient again presented with progressively deteriorating cough in the absence of any change in drug therapy. Physical examination was normal. Laboratory investigations, including arterial blood gases at rest, complete blood count, C reactive protein, and immunoelectrophoresis, were normal. Pulmonary function tests demonstrated an obstructive pattern with an FEV_1_/FVC ratio of 0.40 and FEV_1_ 91% of predicted (1.37 L). Expiratory flow-volume loop showed a reduced peak-flow and a concave appearance of the loop without reversibility after bronchodilators. Inspiratory curve showed a plateau aspect (Figure 1A). CT scan revealed a mass arising from the anterior wall of the trachea that projects into the tracheal lumen without intrathoracic lymphadenopathy (Figure 2). The mass was homogenous without calcification or necrosis. There was paratracheal fat stranding. Fiberoptic bronchoscopy showed a 2 cm hypervascular multilobular mass arising from the anterior tracheal wall, situated 1 cm above the carina at the same site of 1 of the 2 lesions identified in 2010, and causing 50% reduction of the tracheal lumen (Figure 3). Mechanical resection combined with electrocoagulation was performed via rigid bronchoscopy with no complication. Histological examination of the tracheal lesion demonstrated RDD (Figure 4). At 1-year follow-up, patient was symptom free without recurrence of cough and previously altered flow-volume loop had normalized (Figure 1B). Informed consent was signed by the patient.

## DISCUSSION

RDD was initially described by Destombes in 1965^3^ and recognized as a distinct clinicopathological entity by Rosai and Dorfman in 1969.^4^ This disease is characterized histologically by an abnormal proliferation of histiocytes, typically with positive immunolabeling for S-100 protein and CD68, with engulfment of lymphocytes called emperipolesis. Immunohistological staining for CD1a is negative, excluding the diagnosis of Langerhans cell histiocytosis.^5^ The etiology of RDD is unknown, although viral infections or immune dysfunction have been proposed.^6–8^

RDD can occur at any age, but the disease is more common in the first or second decades of life with a mean age at onset of 20.6 years.^1^ Usual signs are painless cervical lymphadenopathy (87.3%) with fever, night sweats, and weight loss.^1,9,10^ Axillary (23.7%), inguinal (25.7%), and mediastinal (14.5%) lymph node groups are commonly affected. Forty-three percent of patients have extranodal involvement, including skin (11.5%), paranasal sinuses (11.3%), upper aerodigestive tract (11.3%), soft tissues (8.9%), eyes (8.5%), bone (7.8%), central nervous system (4.9%), and kidneys (2.3%).^1,11,12^ Intrathoracic manifestations are rarely observed (2%), including pulmonary nodules or masses, interstitial lung disease, central airway involvement, and pleural effusion.^1,2,13–15^ Patients with a chronic aggressive form affecting the kidneys, liver, or lower respiratory tract may have a poorer prognosis.^1^

To our knowledge, only 12 cases of tracheobronchial involvement of RDD, including our case, have been reported in the English language literature^1,13,15–23^ and some of these cases consisted of laryngeal involvement with tracheal extension^16^ (Table 1). These cases were observed in 6 men and 5 women (missing data, n = 1) with a mean age of 40.6 years at the time of diagnosis of airway involvement. In 8 cases, tracheobronchial involvement was the first manifestation of RDD. Acute respiratory failure (n = 4), progressive dyspnea (n = 3), and cough (n = 4) were the main symptoms. Concomitant cervical lymphadenopathy was present in 5 cases. Six patients had extranodal involvement at the time of RDD diagnosis with nasal (n = 3), sinus (n = 1), ear (n = 1), eye (n = 1), and skin (n = 1) manifestations (data not shown). Infiltration of the tracheobronchial tree may be responsible for an obstructive pattern on pulmonary function test.^22^ On fiberoptic bronchoscopy, the size of tracheobronchial lesions ranged from granular infiltration to a 40 mm mass. Of note, subglottic stenosis can also be secondary to extrinsic compression by massive lymphadenopathy.^24^ Increased uptake on positron emission tomography has been reported.^13^ Therefore, in absence of medical history, airway involvement of RDD can mimic tracheal carcinoma, other rare tracheal tumors (including plasmocytoma, melanoma, papilloma), or more rarely granulomatous lesions (as reported in Crohn disease, polyangeitis granulomatosis, or tracheal amyloidosis). Diagnosis can only be established on histological examination.

**TABLE 1:**
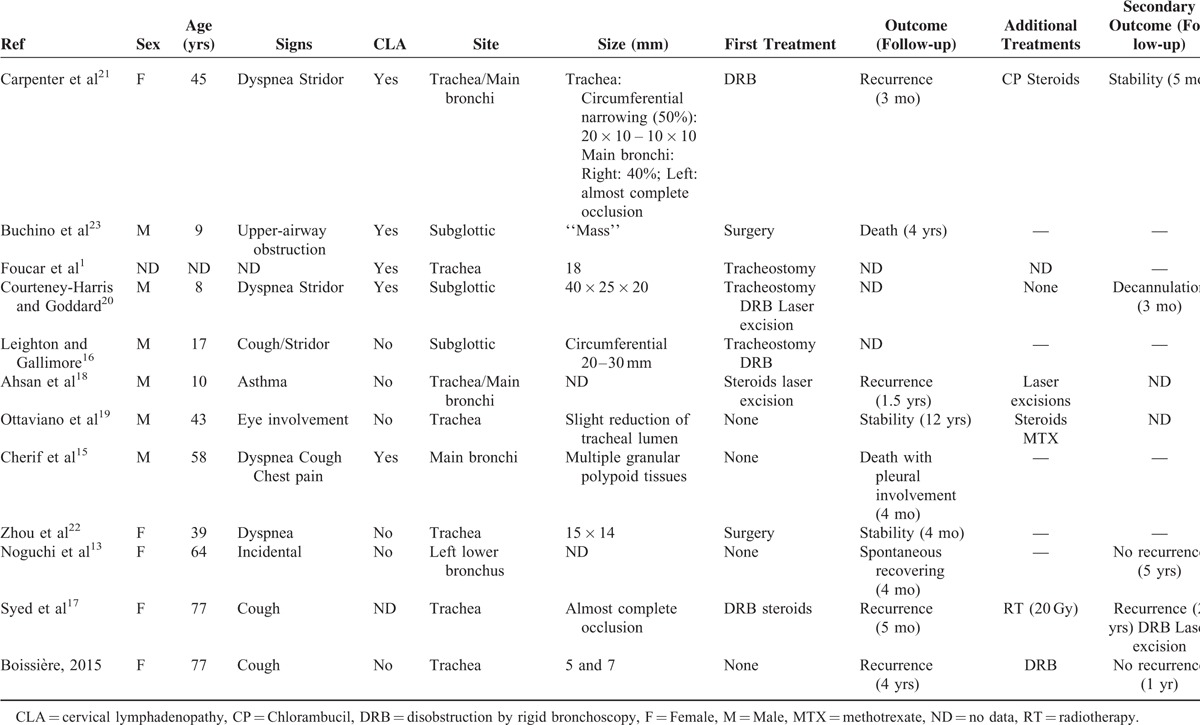
Characteristics, Treatment, and Follow-up Data of the Patients With Tracheo-bronchial Involvement of RDD

The therapeutic management of tracheobronchial RDD required tracheostomy in 3 patients because of acute respiratory failure. Various treatments have been described, including debulking resection by rigid bronchoscopy (n = 4), laser resection (n = 2), or surgery (n = 2). Corticosteroids were used in 2 cases without efficacy, first before laser excision^18^ and second after disobstruction by rigid bronchoscopy.^17^ Watchful waiting was proposed in 3 cases because of incidental discovery. In 1 case, a rapidly fatal outcome did not allow any therapeutic management.^15^ Very few data are available concerning long-term outcome. Tracheobronchial RDD recurrence was observed in several cases. Spontaneous recovery was observed in 1 patient.^13^ Two deaths were reported, 1 related to RDD bronchial and pleural involvement^15^ and 1 due to severe pulmonary edema in a young child with neurological RDD damage.^23^ It is difficult to draw any definite conclusions concerning management of tracheobronchial RDD due to the small number of cases and the limited data on long-term follow-up. In our case, the patient remained asymptomatic for 4 years after the initial diagnosis of tracheal RDD, and then developed a symptomatic tracheal mass, which was successfully resected by rigid bronchoscopy with no recurrence after a follow-up of 1 year. No recommendation has been proposed for systematic bronchoscopy long-term follow-up after RDD disobstruction. Annual clinical assessment and pulmonary function tests may be useful to detect recurrence and to consider the practice of a bronchoscopy.

In conclusion, tracheobronchial involvement of RDD is unusual. Clinical and radiological manifestations can mimic tracheal carcinoma or other rare tumors, consequently requiring careful histological examination. The therapeutic management of tracheobronchial RDD has not been clearly defined, but may require disobstruction by rigid bronchoscopy or surgery in the presence of symptomatic tracheobronchial obstruction. Recurrence is frequent, justifying long-term follow-up.
